# Exercise regulates mitophagy to alleviate parkinsonian neurodegeneration

**DOI:** 10.3389/fnagi.2025.1678460

**Published:** 2025-11-21

**Authors:** Chang Liu, Wei He, JianHua Zhang

**Affiliations:** 1 School of Physical Education and Arts, Hunan University of Medicine, Hunan, China; 2 University of Perpetual Help System DALTA (UPHSD), Las Piñas, Philippines; 3 Xiangzhong Normal College for Preschool Education, Shaoyang, China

**Keywords:** Parkinson’s disease, mitophagy, exercise intervention, AMPK signaling, PINK1/Parkin pathway

## Abstract

Parkinson’s disease (PD) is a common neurodegenerative disorder with a rising incidence in aging populations, substantially diminishing patients’ quality of life. Mitochondria are central to neuronal energy metabolism, and mitophagy plays a pivotal role in maintaining mitochondrial quality by removing damaged organelles. In PD, impaired mitophagy leads to the accumulation of dysfunctional mitochondria, exacerbating oxidative stress and bioenergetic deficits and thereby accelerating disease progression. In recent years, exercise has emerged as a safe and cost-effective intervention that alleviates PD symptoms. Exercise can activate mitophagy through key signaling pathways—including AMP-activated protein kinase (AMPK)/Unc-51–like kinase 1 (ULK1) and PTEN-induced kinase 1 (PINK1)/Parkin—thereby enhancing mitochondrial function and antioxidant capacity. This review synthesizes current evidence on how exercise modulates mitophagy to confer neuroprotection in PD, providing conceptual and practical insights for non-pharmacological management strategies in neurodegenerative disease.

## 1 Introduction

Parkinson’s disease (PD) is a common neurodegenerative disorder characterized pathologically by the progressive loss of dopaminergic neurons in the substantia nigra and other midbrain nuclei (Zhou et al., 2019). In recent years, accumulating evidence has underscored mitochondrial dysfunction and impaired autophagy as central contributors to PD pathogenesis (Chen J. et al., 2018). Beyond supplying energy for neuronal activity, mitochondria play key roles in regulating oxidative stress, calcium homeostasis, and cell death pathways (Okur and Djalilian, 2020; Yañez et al., 2026). As a selective form of autophagy, mitophagy maintains mitochondrial homeostasis by removing damaged or dysfunctional mitochondria, thereby preventing the accumulation of toxic species and further neuronal injury. However, mitophagy is frequently compromised in patients with PD, resulting in defective clearance of abnormal mitochondria and exacerbation of disease progression (Wang M. et al., 2021).

With advances in PD-related molecular research, growing evidence suggests that restoring mitochondrial function and enhancing mitophagy may represent promising strategies for disease prevention and therapy (Gan et al., 2024). As a safe, economical, and readily implementable non-pharmacological intervention, exercise has garnered substantial attention in PD management. An increasing body of clinical and experimental data indicates that regular physical activity not only improves motor symptoms and quality of life in PD, but also confers neuroprotection through multiple mechanisms (Nguyen et al., 2025). These include modulation of key signaling pathways—such as PINK1/Parkin and AMP-activated protein kinase (AMPK)—to promote mitochondrial recovery and activate mitophagy, ultimately supporting neuronal health at multiple levels (Chen C. et al., 2022; Guo et al., 2023; Li et al., 2024).

Accordingly, this review aims to systematically delineate the role and molecular mechanisms by which exercise regulates mitophagy in the context of PD prevention and treatment. We focus on the effects of exercise on mitochondrial function, the autophagic process, and related signaling cascades. The goal is to provide a theoretical foundation and practical guidance for non-pharmacological interventions in PD, thereby informing comprehensive disease management.

## 2 Mitophagy

Mitophagy is a selective form of autophagy that specifically recognizes and eliminates damaged mitochondria, constituting a core component of mitochondrial quality control. Unlike non-selective autophagy, mitophagy exhibits high specificity, targeting dysfunctional or stressed mitochondria for removal through the autophagosome–lysosome pathway (Wang X. et al., 2019). This process is essential for preserving mitochondrial integrity and cellular energy homeostasis. Because neurons rely heavily on mitochondrial oxidative phosphorylation and are particularly vulnerable to oxidative stress, efficient mitophagy is especially critical for neuronal survival. Numerous experimental studies have linked impaired mitophagy to the pathogenesis of neurodegenerative diseases, including Parkinson’s disease (PD) (Antico et al., 2025; Rappe et al., 2024; Wang S. et al., 2023; Xiao et al., 2022; Yang et al., 2025).

**FIGURE 1:**
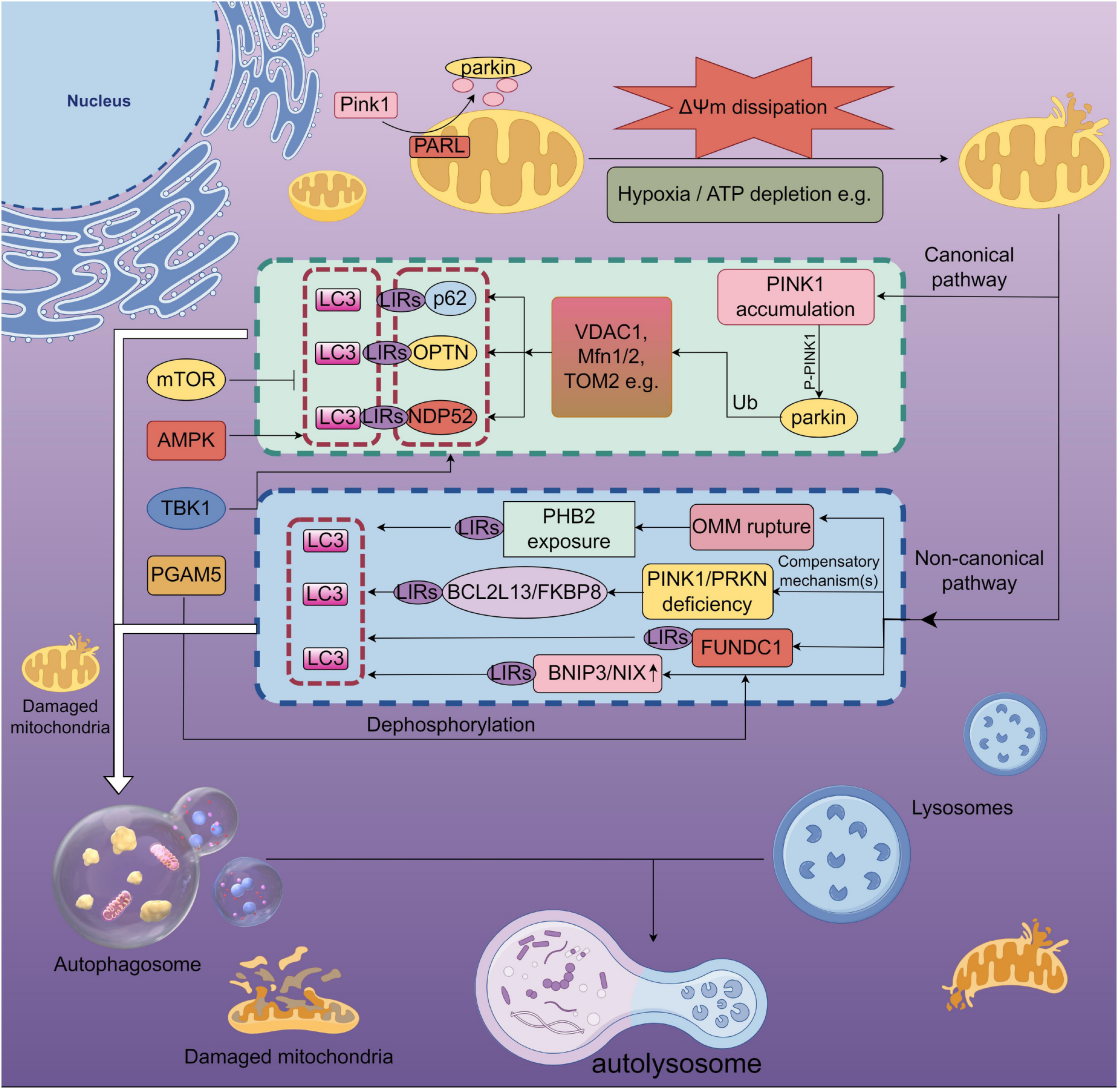
Schematic of mitophagy under exercise-related regulation (By Figdraw). Loss of mitochondrial membrane potential (ΔΨm dissipation) or hypoxia/ATP depletion stabilizes PINK1 on the outer mitochondrial membrane (OMM) by preventing its processing by PARL. PINK1 then phosphorylates ubiquitin and activates Parkin (PRKN), amplifying ubiquitination of OMM proteins such as VDAC1, MFN1/2, and TOM20. These tags recruit the receptors OPTN and NDP52, which use their LIR motifs to bind LC3 and enwrap damaged mitochondria into autophagosomes. On the left, exercise/energy-stress regulators are shown: AMPK promotes mitophagy and counteracts mTOR inhibition, while TBK1 phosphorylation enhances the LC3 binding of OPTN/NDP52. The blue dashed panel depicts receptor-mediated non-canonical routes: dephosphorylated FUNDC1 (facilitated by PGAM5) directly binds LC3; BNIP3/NIX and BCL2L13/FKBP8 can also serve as LC3-interacting receptors; when the OMM ruptures, the inner-membrane protein PHB2 becomes exposed and functions as an LC3 receptor. The lower pathway illustrates flux: autophagosome formation, fusion with lysosomes, and degradation in the autolysosome. The “PINK1/PRKN deficiency” box indicates that receptor pathways may partially compensate when the canonical pathway is impaired. ΔΨm, mitochondrial membrane potential; OMM, outer mitochondrial membrane; Ub, ubiquitin; LC3, microtubule-associated protein 1 light chain 3; LIR, LC3-interacting region; OPTN, optineurin; NDP52, also CALCOCO2; AMPK, AMP-activated protein kinase; mTOR, mechanistic target of rapamycin; TBK1, TANK-binding kinase 1; PARL, presenilin-associated rhomboid-like protease; PGAM5, phosphoglycerate mutase family member 5; BNIP3/NIX, NIP3-like protein X (BNIP3L); FUNDC1, FUN14 domain-containing protein 1; PHB2, prohibitin-2; BCL2L13, BCL-2-like protein 13; FKBP8, FK506-binding protein 8; MFN1/2, mitofusin-1/2; VDAC1, voltage-dependent anion channel 1; TOM20, translocase of the outer mitochondrial membrane 20.

Current evidence indicates that mitophagy is governed by two major classes of signaling mechanisms: (i) a ubiquitin-dependent pathway mediated primarily by PTEN-induced kinase 1 (PINK1) and the E3 ubiquitin ligase Parkin; and (ii) ubiquitin-independent, receptor-mediated pathways involving proteins such as BNIP3, NIX, and FUNDC1 (Lim and Lim, 2017; see Figure 1). Among these, the PINK1/Parkin cascade is the most extensively characterized.

Under physiological conditions, PINK1 is imported into the inner mitochondrial membrane (IMM) and degraded by the mitochondrial protease PARL (Baninameh et al., 2025; Siebert et al., 2022). When the mitochondrial membrane potential (Δψm) collapses, PINK1 accumulates on the outer mitochondrial membrane (OMM), undergoes autophosphorylation, and recruits and activates Parkin (Guardia-Laguarta et al., 2019). Activated Parkin ubiquitinates multiple OMM proteins—including VDAC1, mitofusin-1/2 (Mfn1/2), and TOM20—generating K63-linked polyubiquitin chains that are recognized by autophagy receptors such as p62/SQSTM1, optineurin (OPTN), and NDP52 (Barodia et al., 2019; Bradshaw et al., 2021). These receptors contain LC3-interacting region (LIR) motifs that bind LC3 on nascent autophagosomal membranes, thereby promoting sequestration of damaged mitochondria, followed by lysosomal fusion and degradation (Wang S. et al., 2023). This process is further fine-tuned by regulatory factors including TANK-binding kinase 1 (TBK1), AMP-activated protein kinase (AMPK), and mechanistic target of rapamycin (mTOR) (Belousov et al., 2021).

In parallel, to compensate for PINK1 or Parkin deficiency and under specific stresses, cells deploy several Parkin-independent, receptor-mediated mitophagy routes. Under hypoxia or ATP depletion, BNIP3 and NIX are upregulated and can directly engage LC3 to initiate mitophagy (Yamashita et al., 2024). During hypoxia, FUNDC1 is dephosphorylated by PGAM5, which increases its affinity for LC3; likewise, upon OMM rupture, the IMM protein prohibitin-2 (PHB2) becomes exposed to the cytosol and acts as an LC3 receptor to facilitate degradation of the inner membrane (Chen Z. et al., 2017; Roy et al., 2025). Newly identified receptors, such as BCL2L13 and FKBP8, also exert compensatory roles when Parkin is absent (Bhujabal et al., 2017). Most of these non-canonical receptors harbor LIR motifs and interact with the LC3/GABARAP family to promote the clearance of damaged mitochondria.

In sum, mitophagy is a coordinated, multistep process encompassing damage recognition, ubiquitin tagging, adaptor recruitment, membrane encapsulation, and autophagosome–lysosome fusion and degradation. Together, the PINK1/Parkin axis and diverse non-canonical receptor pathways form a robust and partially redundant network that is indispensable for maintaining mitochondrial homeostasis in neurons (Imberechts et al., 2022). Disruption of this system is a key initiating factor in PD and related disorders (Rai et al., 2024). Elucidating the molecular basis of mitophagy is therefore crucial for understanding disease mechanisms and identifying potential therapeutic targets (Cai and Jeong, 2020; Cen et al., 2021; Shefa et al., 2019).

## 3 Mitophagy impairment and the pathogenesis of PD

Converging evidence indicates that mitophagy is broadly compromised in patients with PD, potentially playing a key role in neurodegeneration (Sharabi et al., 2021). Postmortem analyses reveal abnormal accumulation of the outer-mitochondrial-membrane (OMM) small GTPase Miro—an adaptor for mitochondrial transport and a degradation substrate during PINK1/Parkin-mediated mitophagy—suggesting defective clearance and impaired quality control; this alteration is absent in age-matched controls (Drwesh et al., 2025; Gao et al., 2020; Hsieh et al., 2016). Consistently, platelets from patients with PD display marked reductions in autophagy markers such as LC3-II and MsrB2 (Lee et al., 2019). In induced pluripotent stem cell (iPSC)-derived dopaminergic (DA) neurons—including models carrying the LRRK2 G2019S mutation and those derived from sporadic PD—mitochondrial depolarization fails to trigger proper Miro degradation, thereby disrupting the recruitment of the Parkin-dependent autophagic machinery (Bharat and Wang, 2020; Hsieh et al., 2016). Neurons expressing mutant forms of α-synuclein (α-syn) similarly exhibit Miro accumulation and mitophagy defects (Xiao et al., 2022). Collectively, cytological observations and genetic data support a pathogenic link between impaired mitophagy and PD, with genotype- and phenotype-specific effect sizes. Pathogenic variants in PINK1 (a mitochondrial serine/threonine kinase) and PRKN (encoding the E3 ubiquitin ligase Parkin) cause subsets of familial PD and directly disrupt damage recognition and clearance. By contrast, LRRK2 G2019S–associated mitophagy defects can be partially rescued by LRRK2 kinase inhibitors but not in PINK1/PRKN deficiency, implying the need for etiologically stratified therapeutic strategies (Barodia et al., 2017; Bonello et al., 2019; Narendra et al., 2013).

### 3.1 Oxidative stress and mitophagy in PD

Reactive oxygen species (ROS) are markedly elevated in the PD brain. Major sources include dopamine auto-oxidation—which generates quinones and superoxide (O_2_^•−^)—and high iron content in the substantia nigra (SN), which catalyzes hydroxyl radical (•OH) formation via the Fenton reaction (Martinez and Peplow, 2017; Teerapattarakan et al., 2018). Dysfunction of respiratory chain complexes I and III further promotes electron leak and ROS production. These factors converge to collapse the mitochondrial membrane potential (Δψm) and disrupt inner mitochondrial membrane (IMM) architecture (Dias et al., 2023).

Intriguingly, moderate ROS can act as signaling cues, activating stress-response pathways. ROS stimulate the AMPK–mTORC1 axis and the JNK pathway, both of which enhance PINK1/Parkin-dependent mitophagy, thereby facilitating removal of damaged mitochondria and attenuating DA neuron apoptosis (Park et al., 2017; Xiao et al., 2017). In the MPTP mouse model, either moderate exercise or treatment with N-acetylcysteine (NAC) lowers ROS levels in the SN, increases LC3-II expression, and augments Parkin recruitment to mitochondria; these interventions improve motor performance and enhance DA neuron survival (Hwang et al., 2018; Monti et al., 2016).

In contrast, chronically elevated or excessive ROS inflict irreversible damage on the autophagy–lysosome system. Overabundant ROS oxidize key autophagy proteins (e.g., ATG3 and ATG7), inhibiting the lipidation of LC3-I to LC3-II (Zhou et al., 2022). Oxidative injury to lysosomal membrane proteins (e.g., LAMP2) blocks autophagic flux and causes p62 accumulation (Wang X. et al., 2019). In neuronal models exposed to 6-hydroxydopamine (6-OHDA), high ROS impairs mitophagy and exacerbates apoptosis (Di Rita et al., 2018).

Taken together, these data support a “dose–response” paradigm: moderate ROS promote neuroprotection by activating AMPK–mTORC1 and JNK signaling to facilitate PINK1/Parkin-mediated mitophagy, whereas chronic/excess ROS oxidatively disable ATG3/ATG7 and LAMP2, disrupt autophagic flux, drive accumulation of dysfunctional mitochondria, and accelerate neurodegeneration. Future work should define quantitative thresholds and molecular determinants of ROS–mitophagy coupling and develop combination strategies that both mitigate oxidative stress and precisely activate PINK1/Parkin-dependent mitophagy, with the goal of disease modification in PD.

### 3.2 α-synuclein aggregation and mitophagy

Misfolding and aggregation of α-synuclein (α-syn) are pathological hallmarks of PD. Mutations or copy-number gains in SNCA cause α-syn overexpression and Lewy body formation in neurons (Polymeropoulos et al., 1997). Aggregated α-syn impairs mitophagy via two principal routes. First, pathological α-syn anchors to the OMM and gains access through the translocase of the outer membrane (TOM) complex, perturbing mitochondria–ER contact sites (MAMs) and disrupting calcium homeostasis (Paillusson et al., 2017). Oligomeric α-syn further inhibits complex I activity, reduces ATP production, increases electron leak and ROS generation, collapses Δψm, and opens the mitochondrial permeability transition pore (mPTP), collectively activating mitochondrial apoptosis and triggering PINK1/Parkin-dependent mitophagy to clear damaged organelles (Reeve et al., 2015).

Second, abundant evidence indicates that α-syn aggregation not only injures mitochondria but also compromises the execution of mitophagy itself. Lewy bodies from PD brains contain not only α-syn aggregates but also mitochondrial membrane components and autophagolysosomal remnants, suggestive of stalled flux (Lenzi et al., 2024). In Drosophila, cultured neurons, and human iPSC-derived DA neurons, α-syn overexpression preferentially suppresses PINK1/Parkin-mediated mitophagy while sparing bulk, non-selective autophagy (Kinnart et al., 2024). Although PINK1 and Parkin are recruited to depolarized mitochondria, downstream steps—phagophore formation, sequestration of damaged mitochondria, trafficking, and lysosomal fusion—are impeded, resulting in impaired mitophagy flux.

Mechanistically, excessive α-syn promotes and stabilizes hyperpolymerization of the actin cytoskeleton, reducing the plasticity of autophagosomal membranes and their capacity to engulf damaged mitochondria (Kinnart et al., 2024; Lurette et al., 2023). Overexpressing the actin-severing protein cofilin, or pharmacologically inhibiting the Arp2/3 complex to restore actin depolymerization, partially rescues mitophagy flux and mitochondrial clearance (Sarkar et al., 2021). Moreover, pathogenic α-syn variants interfere with Parkin-mediated ubiquitination of Miro, preventing its degradation; damaged mitochondria remain tethered to microtubules, hindering autophagosome recruitment and initiation of mitophagy (D’Errico et al., 2021).

Summary. Pathological α-syn both aggravates mitochondrial damage and disrupts multiple components of the mitophagy machinery, creating a vicious cycle of “mitochondrial dysfunction → defective clearance → further damage” that accelerates DA neuron loss. Elucidating how α-syn modulates TOM complex function, MAM integrity, actin remodeling, and Miro degradation may enable targeted strategies to counter α-syn aggregation or its inhibitory pathways and restore mitophagy balance.

### 3.3 Neuroinflammation and mitophagy in PD

Chronic neuroinflammation is a prominent feature of PD. Activated microglia and astrocytes release proinflammatory mediators—including IL-1β, TNF-α, IL-6, and nitric oxide (NO)—which exacerbate DA neuron injury and apoptosis (Chen G. et al., 2017; Guo et al., 2025). Within this inflammatory milieu, upstream stimuli such as ROS activate NF-κB signaling. NF-κB not only drives classical inflammatory gene expression but also upregulates autophagy-related factors, including the receptor p62 and PINK1, thereby enhancing Parkin-dependent mitophagy, constraining NLRP3 inflammasome overactivation, and limiting mitochondrial dysfunction (Song et al., 2020; Zhong et al., 2016).

Notably, inhibition of NF-κB–p62–driven mitophagy aggravates programmed cell death in inflammatory macrophages, underscoring a “dual role” for NF-κB in balancing inflammation via mitophagy regulation (Yuk et al., 2020). Conversely, chronic neuroinflammation is often accompanied by sustained activation of mTOR signaling and cumulative oxidative stress, which suppress expression of the autophagy-initiating kinase ULK1. Although Beclin 1 may be upregulated as compensation, autophagic flux remains insufficient to restore homeostasis, leading to inadequate clearance of damaged proteins and organelles, α-syn accumulation, and heightened neurotoxicity (Hosokawa et al., 2009; Spencer et al., 2009).

Mitophagy defects can further amplify neuroinflammation in a feed-forward manner. In mice with microglia-specific Atg5 deletion, α-syn overexpression increases proinflammatory cytokine release and exacerbates DA neuron loss (Qin et al., 2021). Likewise, in peripheral immune cells of patients with PD, impaired mitophagy allows damaged mitochondria and their damage-associated molecular patterns (DAMPs)—including mitochondrial DNA and ROS—to accumulate, activating the NLRP3 inflammasome and promoting IL-1β secretion (Tansey et al., 2022). Encouragingly, enhancing general autophagy or selective mitophagy facilitates the clearance of dysfunctional mitochondria and protein aggregates, suppresses NLRP3 assembly and activation, mitigates neuroinflammation, and supports DA neuron survival (Kinet and Dehay, 2023).

Summary. Neuroinflammation and mitophagy are tightly interlinked through complex positive and negative feedback loops. Moderate activation of mitophagy can buffer inflammatory signaling, whereas impaired mitophagy aggravates chronic inflammation and drives neurodegeneration.

### 3.4 Calcium dyshomeostasis and mitophagy in PD

Calcium (Ca^2+^) is essential for the autonomous pacemaking and synaptic signaling of DA neurons, largely due to sustained Ca^2+^ influx through L-type voltage-gated calcium channels, particularly CaV1.2 and CaV1.3 (Liu et al., 2014). When mitochondria are intact, excess cytosolic Ca^2+^ is rapidly sequestered into the matrix and extruded via Ca^2+^ pumps and Na^+^/Ca^2+^ exchangers to maintain homeostasis. In PD, mitochondrial Ca^2+^ buffering is impaired. In addition, α-syn aggregation and related factors disrupt MAMs, perturbing Ca^2+^ uptake and release during cytosolic transients and resulting in Ca^2+^ overload in both the cytosol and mitochondria (Henrich et al., 2023; Paillusson et al., 2017).

Beyond its canonical role in mitochondrial quality control, PINK1 maintains Ca^2+^ homeostasis by promoting mitochondrial Ca^2+^ efflux. In PINK1-deficient models, matrix Ca^2+^ accumulates, triggering mPTP opening, Δψm loss, mitochondrial swelling, cytochrome c release, and DA neuron apoptosis (Gandhi et al., 2009; Gautier et al., 2012). Using mitochondria-targeted photosensitizers to induce localized matrix Ca^2+^ oscillations, studies show that even physiological Ca^2+^ transients can stabilize PINK1 accumulation on the OMM and recruit Parkin, thereby initiating selective mitophagy without global mitochondrial depolarization (Tian and He, 2022; Yu et al., 2021). Moreover, the Ca^2+^-dependent phosphatase calcineurin promotes Parkin translocation to damaged mitochondria via dephosphorylation and activates transcription factors such as TFEB, linking Ca^2+^ signaling to mitophagy at both post-translational and transcriptional levels (Marchesan et al., 2024; Tong and Song, 2015).

Nevertheless, under PD-related pathological conditions, sustained Ca^2+^ overload causes excessive mPTP opening and copious ROS generation, pushing mitochondria into irreversible failure before timely clearance can occur. Dysregulated Ca^2+^ signaling may either blunt proper activation of PINK1/Parkin-mediated mitophagy or, conversely, drive its overactivation and eventual exhaustion, further disrupting mitochondrial clearance (Xiao et al., 2022).

Summary. Calcium imbalance in PD not only worsens mitochondrial injury but also perturbs Ca^2+^-dependent signaling that governs mitophagy, jointly accelerating DA neurodegeneration. Ca^2+^ thus serves as both trigger and modulator of mitophagy in PD pathophysiology.

### 3.5 Ferroptosis and mitophagy

Iron dyshomeostasis is a key pathological feature of PD, with significantly elevated iron in the SN (Faucheux et al., 2003). Excess ferrous iron (Fe^2+^) catalyzes the Fenton reaction, converting H_2_O_2_ into highly reactive ∙OH radicals, thereby intensifying oxidative stress. This promotes lipid peroxidation and protein oxidation of mitochondrial membranes, ultimately compromising mitochondrial function (Jomova et al., 2010). Iron can also interact directly with α-syn, promoting conformational rearrangement and oligomerization, enhancing neurotoxicity, and further escalating ROS production and mitochondrial dysfunction (Deas et al., 2016).

Iron overload triggers ferroptosis—a regulated cell death modality characterized by cristae collapse and lipid peroxide accumulation—which rapidly increases the burden of damaged mitochondria and the demand on the mitophagy system (Li et al., 2022). However, when iron accumulates within lysosomes, it impairs v-ATPase–mediated acidification and inhibits autophagosome–lysosome fusion and degradative activity. As a result, damaged mitochondria and misfolded protein aggregates are inefficiently cleared, establishing a vicious cycle of “iron dyshomeostasis → mitophagy dysfunction” (Jahng et al., 2019). As mitophagy efficiency declines, damaged mitochondria continue to release ROS and proinflammatory mediators, amplifying neuroinflammation and cellular stress and accelerating progressive DA neuron loss (Xu J. et al., 2025).

Summary. In PD, iron overload amplifies ROS via the Fenton reaction, drives lipid peroxidation, and synergizes with α-syn to damage mitochondria and induce ferroptosis. Lysosomal iron further compromises acidification and autophagic degradation, impeding mitochondrial clearance. Reducing iron burden and restoring mitophagy/lysosomal function are therefore pivotal to breaking this cycle.

### 3.6 PD genes and mitophagy

Beyond the canonical PINK1/PRKN axis, an expanding roster of PD risk and causal genes converge on mitophagy regulation (Table 1), impeding three functional tiers: (i) damage tagging and receptor recruitment, (ii) dynamical pre-processing (fission/fusion and segregation), and (iii) autophagolysosomal degradation. For example, hyperactive LRRK2 (e.g., G2019S) overphosphorylates Rab GTPases, weakening the localization of receptors such as OPTN on damaged mitochondria and suppressing Parkin-dependent tagging and clearance (Wauters et al., 2020). GBA1 deficiency is marked by lysosomal enzyme insufficiency and mTORC1–TFEB imbalance, creating a “flux bottleneck” at the post-fusion degradative step; mitophagy inhibition feeds back to α-syn aggregation (Perez-Abshana et al., 2025). DJ-1 (PARK7), a redox sensor positioned at the PINK1/PRKN interface, does not prevent PINK1 stabilization or Parkin activation but critically promotes assembly/enrichment of receptors such as OPTN/NDP52; loss causes selective mitophagy stalling (Sarkar and Singh, 2024). VPS35 (retromer core) maintains fission/fusion balance via DRP1 cycling and the MUL1–MFN2 axis; mutations (e.g., D620N) induce dynamical imbalance that hampers segregation of damaged fragments into the autophagic pathway and reduces PINK1/PRKN-mediated clearance efficiency (Ma et al., 2021).

**TABLE 1:** Parkinson’s disease (PD)-associated genes impacting mitophagy at tagging, dynamics, and degradation steps.

Gene	Alias/locus	Primary step in mitophagy	Key mechanistic points	Effect on mitophagy	Evidence/Risk in PD	References
DJ-1	PARK7	Tagging/receptor recruitment	Redox sensor; helps relay Parkin signaling to receptors such as OPTN/NDP52; oxidative-stress responsive	Defective assembly/selection of receptors when DJ-1 is lost, stalling selective mitophagy	Pathogenic variants cause familial PD; loss of antioxidant defense impedes damaged-mitochondria clearance	Imberechts et al., 2022
LRRK2	PARK8; G2019S, etc.,	Tagging upstream; Rab-GTPase regulation	Hyperactive kinase over-phosphorylates Rabs (e.g., Rab10), weakens localization of OPTN and related receptors on damaged mitochondria	Decreased mitophagy; accumulation of dysfunctional mitochondria	Familial PD mutations common; associated with Lewy pathology	Pang et al., 2022
VPS35	Retromer core; D620N	Dynamical pre-processing (fission/fusion segregation)	Alters DRP1 cycling and MUL1–MFN2 axis; disrupts mitochondrial dynamics	Harder to segregate damaged fragments into the autophagic route; reduced PINK1/PRKN-mediated clearance efficiency	Familial PD mutation; linked to mitochondrial damage	Shiraishi et al., 2024
GBA	GBA1; GCase	Autophagolysosomal degradation	Glucocerebrosidase deficiency → lysosomal dysfunction; mTORC1–TFEB imbalance	Post-fusion “flux bottleneck”; accumulation of damaged mitochondria; positive feedback to **α-syn** aggregation	Most important genetic risk factor for PD; accelerates disease progression	Senkevich and Gan-Or, 2020
ATP13A2	PARK9	Lysosomal ion/pH homeostasis (degradation arm)	Loss of function causes abnormal lysosomal pH and ion imbalance	Reduced fusion/degradation efficiency; impaired mitophagy	Early-onset PD; autophagy–lysosome dysfunction	Johnson et al., 2021
SYNJ1	Synaptojanin-1	Membrane sourcing and autophagosome maturation	Endocytic defects limit membrane supply and autophagosome formation	Mitochondrial degradation impaired; mitophagy flux reduced	Early-onset PD; mitochondrial quality control affected	Nguyen et al., 2019
DNAJC6	Auxilin	Clathrin uncoating/vesicle trafficking (membrane supply)	Perturbs clathrin-coated vesicle cycle; interferes with autophagosome membrane sourcing	Indirect inhibition of mitophagy; decreased throughput	Early-onset PD–related; synaptic endocytosis defects	Nguyen et al., 2019
SH3GL2	Endophilin-A1	Endocytosis/vesicle trafficking → autophagosome formation	Membrane curvature/scission abnormalities; autophagosome biogenesis less efficient	Lower mitophagy efficiency; reduced degradative capacity	PD risk locus; linked to autophagy and mitochondrial injury	Senkevich and Gan-Or, 2020
USP24	Deubiquitinase	Signaling layer upstream of cargo tagging	Excess deubiquitination counteracts Parkin-driven ubiquitin tagging	Negative regulator of selective mitochondrial clearance	Located at PD susceptibility regions; acts as autophagy brake	Thayer et al., 2020
TMEM175	Lysosomal K + channel	Autophagolysosomal degradation (acidification/voltage)	Channel defects impair lysosomal membrane potential and acidification; disturbed ionic homeostasis	Inefficient cargo degradation; promotes **α-syn** accumulation; reduced ALP capacity	Increases PD risk; compromises mitochondrial quality control	Johnson et al., 2021

Additional genes converge on the degradative arm or membrane supply: ATP13A2 and TMEM175 compromise lysosomal acidification and ionic homeostasis (Bento et al., 2016; Jinn et al., 2017); SYNJ1, DNAJC6, and SH3GL2 (endocytosis/vesicle-trafficking factors) impact autophagosome membrane sourcing and maturation (Saenz et al., 2024; Weissbach et al., 2019; Wittke et al., 2021); deubiquitinases such as USP24 can negatively tune upstream signaling (Young et al., 2024). In sum, these loci enter via distinct molecular “gateways” yet converge on the same outcome—blocking mitophagy at one or more nodes of the “tag–segregate–degrade” continuum—thus explaining how PD’s polygenic architecture coalesces into mitochondrial quality-control failure.

Overall summary. In PD, oxidative stress, α-syn aggregation, neuroinflammation, Ca^2+^ dyshomeostasis, and iron overload amplify one another to drive mitochondrial injury while suppressing mitophagy at critical stages—from tagging/receptor recruitment to dynamical pre-processing and autophagolysosomal degradation—forming a self-reinforcing loop that propels disease progression. In parallel, a multigenic susceptibility background further weakens this clearance system, enabling long-term accumulation of damaged mitochondria and ROS, fostering protein aggregation and inflammatory spread, and accelerating DA neuron loss. Therapeutic strategies should therefore combine reduction of oxidative and iron burdens, mitigation of inflammation and Ca^2+^ imbalance, restoration of lysosomal function, and targeted enhancement of mitophagy—by activating PINK1/PRKN, optimizing receptor recruitment, correcting mitochondrial dynamics, and promoting TFEB-driven autophagy–lysosome biogenesis. When integrated with an appropriate exercise prescription, such approaches may interrupt the early “injury → clearance failure → further injury” cycle and deliver disease-modifying benefits.

## 4 Exercise and mitophagy

### 4.1 Acute effects of exercise on mitophagy

A single bout of high-intensity exercise can rapidly activate mitophagy in the central nervous system (CNS). Exercise-induced AMPK phosphorylation directly activates ULK1 [e.g., phosphorylation at Ser555 (Guan et al., 2024; Longo et al., 2024)] while relieving its inhibition by suppressing Raptor, a component of mTORC1 (Huang et al., 2021), thereby synergistically promoting the initiation of autophagy. This process is accompanied by an increase in the autophagy marker LC3-II (Wang et al., 2022) and transiently enhances neuronal autophagic activity, likely facilitating autophagosome formation via ULK1 downstream effectors such as BECN1 (Shen et al., 2021), thereby maintaining mitochondrial homeostasis and neural function.

Meanwhile, acute exercise–induced ROS promote the accumulation of PINK1 on the outer mitochondrial membrane and facilitate the recruitment of Parkin, enabling damaged mitochondria to be efficiently tagged and cleared through the autophagosome–lysosome pathway (Chen C. C. W. et al., 2018). Swimming and treadmill models likewise show that in peripheral tissues such as liver and skeletal muscle, acute exercise activates mitophagic flux, characterized by increased phosphorylation of mitochondrial AMPK and the accumulation of p62 on mitochondria. Interestingly, this response appears to be only weakly dependent on PINK1, suggesting the presence of parallel exercise-induced signaling pathways (McCoin et al., 2022).

In addition, in neural injury models, short-term forced exercise accelerates the mitophagic clearance of damaged mitochondria via the STING/TBK1 signaling axis, thereby reducing cellular stress and stabilizing neuronal function (Adriaenssens et al., 2024; Yang et al., 2024). Taken together, these studies indicate that acute exercise rapidly activates mitophagy through multiple converging pathways—including AMPK–mTORC1 and PINK1/Parkin—to eliminate dysfunctional mitochondria and limit excessive ROS accumulation, providing a molecular basis for the neuroprotective effects of exercise under conditions of acute PD-related stress.

### 4.2 Adaptive regulation of mitophagy by long-term exercise

Prolonged, regular exercise coordinates mitochondrial biogenesis, fusion–fission dynamics, and autophagic clearance to enhance mitochondrial quality control and sustain mitochondrial homeostasis in neurons. In a 6-OHDA–induced PD mouse model, Abrishamdar et al. (2023) implemented 4 weeks of aerobic or resistance training (3 days/week, 40 min/day) and observed robust upregulation of the biogenesis markers PGC-1α, NRF-1/2, and TFAM in the substantia nigra; in parallel, the fusion proteins optic atrophy-1 (OPA1; inner-membrane remodeling) and mitofusin-2 (MFN2; OMM fusion) were increased, whereas the fission GTPase dynamin-related protein-1 (Drp1) normalized toward control levels (Jahangiri et al., 2025). These molecular changes synergistically enhanced biogenesis and dynamics, yielding greater mitochondrial abundance and improved respiratory capacity.

Similarly, in the MPTP model, Jang et al. (2018) reported that 6 weeks of treadmill running (5 days/week, 60 min/day) increased NRF-1 and TFAM expression, further elevated OPA1 and MFN2, and effectively modulated Drp1. This adaptive response optimized the fusion–fission balance, accelerated regeneration of functional mitochondria, and supported dopaminergic neuroprotection.

Long-term exercise also activates PINK1/Parkin-dependent mitophagy to facilitate clearance of accumulated damaged mitochondria. In sedentary PD models, p62, PINK1, and Parkin often accumulate on dysfunctional mitochondria, indicating impaired flux (Chen C. et al., 2022; Gu et al., 2024). After 6–8 weeks of treadmill training, these substrates decline markedly, while lysosomal proteins such as LAMP2 and cathepsin L are upregulated (Nhu et al., 2021), consistent with restoration of autophagosome–lysosome fusion and degradative capacity. This adaptive tuning lowers mitochondrial ROS production, suppresses α-synuclein aggregation, and limits pro-apoptotic signaling, thereby improving DA-neuron survival at cellular and organismal levels (Gao B. et al., 2025; Tung et al., 2024; Wang W. et al., 2021).

Clinical data further support neuroprotection from sustained exercise. In patients with PD, 12 weeks of moderate-intensity aerobic training significantly increased plasma antioxidant enzymes [e.g., catalase (CAT), glutathione (GSH)] and reduced oxidative-stress markers [e.g., malondialdehyde (MDA), uric acid] (Tsai et al., 2025). Likewise, 8 weeks of progressive resistance training increased skeletal-muscle superoxide dismutase (SOD) and glutathione peroxidase (GSH-Px) activities by ∼10%, while decreasing MDA and H2O2 levels by ∼15%–16% (Powers and Jackson, 2008). These findings suggest that resistance training enhances antioxidant defenses and attenuates oxidative stress; however, its direct effects—beneficial or detrimental—on mitophagy per se require more definitive evidence. Animal and cell studies imply that resistance-type loading modulates flux via TFEB-mediated autophagy–lysosome pathways and PINK1/PRKN tagging, but direct human/clinical confirmation remains limited and warrants controlled trials with tracer-based verification (Cao et al., 2025; Díaz-Castro et al., 2024; Hentilä et al., 2018; Moradi et al., 2024).

Additional clinical evidence focused on mitochondrial function is accumulating: a 16-week high-intensity combined-training intervention increased skeletal-muscle complex I/IV activities by ∼45%–56% and 39%–54%, respectively, with citrate synthase unchanged—suggesting qualitative functional gains rather than increased mitochondrial content (Kelly et al., 2014). Another 3-month aerobic-plus-strength program improved clinical scores and whole-body metabolism in PD, and ∧31P-MRS phosphocreatine (PCr) recovery correlated with performance/metabolic indices; however, the most pronounced PCr improvements occurred in healthy controls, implying partially constrained mitochondrial adaptation in PD muscle (Krumpolec et al., 2017). Overall, direct human evidence demonstrating “exercise → increased mitophagy flux and improved neural tissue mitochondrial function” remains limited; an ongoing randomized controlled trial (the PARKEX protocol) will evaluate mitochondrial respiration and related endpoints to further test exercise effects on PD mitochondrial function (Magaña et al., 2023). Hence, clinical conclusions should remain cautious, and future PD studies should incorporate objective readouts such as ∧31P-MRS, platelet or PBMC respiration, respiratory-chain activities, and mitophagy/autophagy-flux markers.

In summary, long-term exercise adaptively enhances mitophagy and antioxidant capacity, stabilizes neuronal bioenergetics, and mitigates PD pathology at multiple levels.

### 4.3 Modality-specific regulation of mitophagy by exercise

Evidence from PD-related studies indicates that different exercise modalities emphasize complementary segments of the “initiation–flux–clearance” chain. Aerobic training and high-intensity interval training (HIIT) preferentially amplify front-end events on acute timescales—energy stress triggers AMPK phosphorylation, relieves mTORC1-mediated ULK1 inhibition, and promotes ULK1–Atg13–FIP200 assembly, while a tempered ROS pulse stabilizes PINK1 on the OMM and recruits Parkin. Together with the TBK1–p62/NDP52/OPTN adaptor axis, these pathways achieve precise tagging and sequestration of damaged mitochondria (Guo et al., 2023; Liu and Pan, 2019; Sharma et al., 2023). After multi-week interventions, this front-end advantage consolidates as higher autophagic/mitophagic flux, often coupled to optimized mitochondrial dynamics (OPA1/MFN2 upregulation, DRP1 normalization), thereby promoting biogenesis and functional recovery (Hatsuda et al., 2023; Laker et al., 2017; Lu et al., 2022). By contrast, resistance training tends to strengthen mid- to late-stage flux and clearance: through Akt–mTOR/FoxO3a, it balances substrate mobilization and proteostasis, drives TFEB nuclear translocation and activation of the CLEAR network, and upregulates lysosomal components (e.g., LAMP family proteins, cathepsins) to boost autophagosome–lysosome fusion and cargo degradation—correcting sedentary/pathological bottlenecks and p62 accumulation. Over weeks, both modalities synergize with the PGC-1α–NRF-1/2–TFAM biogenesis axis and OPA1/MFN2/DRP1 re-tuning, yielding increased mitochondrial content and respiratory capacity, reduced oxidative/proteotoxic stress, and stabilization of DA-neuron function (Cao et al., 2025; D’Lugos et al., 2018; Zeng et al., 2020).

It must be emphasized that no published clinical intervention in PD has yet directly quantified an exercise-induced increase in mitophagy *in vivo* with positive results; current conclusions derive largely from animal models, peripheral tissues, or indirect correlates. Within this evidence framework, combined prescriptions (aerobic/HIIT plus resistance) are more likely than single-modality programs to produce a closed-loop gain—front-end “ignition” (AMPK–ULK1, PINK1/PRKN) together with back-end “flux consolidation” (TFEB–CLEAR, lysosomal function) (Bai et al., 2017; Chen C. et al., 2022; Colleluori et al., 2019). As a practical guideline: center the program on moderate-to-moderately-high-intensity aerobic/interval work, complemented by resistance training 2–3 times per week, with intensity and volume individualized for tolerance and safety.

## 5 Mechanisms by which exercise-regulated mitophagy ameliorates PD pathology

Parkinson’s disease (PD) is a neurodegenerative disorder whose pathogenesis involves multiple pathological processes, including oxidative stress, α-synuclein (α-syn) aggregation, neuroinflammation, dysregulation of calcium homeostasis, and ferroptosis (Di Martino et al., 2021; Huang et al., 2023). Emerging evidence indicates that exercise—a safe, low-cost, non-pharmacological intervention—confers neuroprotection by upregulating neurotrophic factors, attenuating oxidative stress, and enhancing mitochondrial function (Li et al., 2023), and is therefore considered an effective strategy in PD management. Of note, exercise can activate mitophagy through multiple signaling pathways, thereby mitigating PD-related pathologies with disease-modifying potential. Treadmill/endurance training upregulates PINK1/Parkin, elevates LC3-II, reduces p62 accumulation, and restores mitochondrial membrane potential across PD models (Nakahira et al., 2015; Nhu et al., 2021); high-intensity interval or aerobic training can enhance TFEB-mediated autophagy–lysosome biogenesis and flux (Zhang et al., 2022); and exercise upregulates SIRT3 to facilitate removal of damaged mitochondria and modulate the NLRP3 inflammasome (Luthra et al., 2025). Together, these findings support the biological plausibility and translational promise of the “exercise–mitophagy” axis in PD.

### 5.1 How exercise-activated mitophagy alleviates oxidative stress in PD

Oxidative stress—a core driver of PD—is largely fueled by sustained ROS production from dysfunctional mitochondria, disrupting redox homeostasis. As a key component of mitochondrial quality control, mitophagy selectively removes damaged mitochondria, halting persistent ROS generation. Recent work shows that exercise activates multiple mitophagy-linked pathways, strengthening the process at several stages and thereby reducing PD-related oxidative damage at the molecular level.

First, exercise-induced energy stress potently activates AMP-activated protein kinase (AMPK), a central metabolic sensor and upstream regulator of mitophagy initiation. During physical activity, rapid ATP consumption and a rising AMP/ATP ratio trigger Thr172 phosphorylation and activation of AMPK (Chen et al., 2003; Richter and Ruderman, 2009). Activated AMPK phosphorylates ULK1 at Ser317 and Ser777, counteracting mTORC1 inhibition and promoting assembly of the ULK1–Atg13–FIP200 initiation complex to start phagophore formation (Kim et al., 2011). AMPK further tunes autophagic flux by regulating ATG proteins and LC3 lipidation, ensuring continuous and efficient processing of damaged mitochondria (Fujita et al., 2008; Nath et al., 2014). In a systematic review, Nhu et al. (2021) reported that treadmill exercise in PD animal models significantly upregulated PINK1, Parkin, and LC3-II while lowering p62, thereby accelerating mitochondrial clearance and reducing ROS accumulation (Wang S. et al., 2023).

Second, the PINK1/Parkin pathway is pivotal to exercise-induced mitophagy. By sensing loss of mitochondrial membrane potential, this cascade recruits Parkin to ubiquitinate OMM substrates and mark organelles for degradation. In MPTP mice, Hwang et al. showed that treadmill exercise reduced abnormally elevated PINK1/PRKN, p62, and LC3-II/I while increasing LAMP2 and cathepsin L, indicating restoration of PINK1/PRKN-linked mitophagy flux and lysosomal function, with consequent reduction of damaged-mitochondria burden and neuroprotection (Hwang et al., 2018).

Third, exercise enhances terminal degradation by activating transcription factor EB (TFEB), the master regulator of the autophagy–lysosome system. Upon nuclear translocation, TFEB induces lysosomal gene programs (e.g., LAMP2, CTSD), promotes autophagosome–lysosome fusion, and ensures efficient degradation of mitochondrial cargo (Yang et al., 2023). Exercise has been shown to promote TFEB nuclear entry, preventing retention of incompletely degraded mitochondria and secondary ROS release, thereby interrupting positive feedback loops of oxidative stress (Palmieri et al., 2011).

Summary. By co-activating AMPK–ULK1, PINK1/Parkin, and TFEB, exercise enhances mitophagy from recognition and initiation through lysosomal degradation, effectively alleviating oxidative stress in PD and offering a mechanistic framework for mitophagy-targeted non-pharmacological interventions.

### 5.2 How exercise-activated mitophagy mitigates α-synuclein aggregation in PD

Aberrant folding and aggregation of α-syn are signature lesions in PD. Intracellular accumulation not only injures mitochondria but also impairs autophagic flux, suppressing mitophagy and creating a damaging feedback loop that intensifies mitochondrial dysfunction and neurotoxicity (Zhao et al., 2023). Recent studies indicate that exercise strengthens recognition and removal of damaged mitochondria—together with α-syn complexes bound on their surface—thereby restricting pathological spread and reducing the toxic burden.

Exercise first activates the AMPK–ULK1 axis to initiate mitophagy: activity-induced energy stress (ATP depletion and increased AMP/ATP) phosphorylates and activates AMPK (Thr172), which suppresses mTORC1 and activates ULK1 to drive phagophore formation (Laker et al., 2017). This pathway facilitates elimination of damaged mitochondria and aids recognition of α-syn aggregates tethered to the OMM (Amireddy et al., 2023; Kim et al., 2025). In parallel, exercise upregulates the deacetylase SIRT1 and activates PGC-1α, which supports mitochondrial dynamics and stabilizes the PINK1/Parkin pathway (Vainshtein and Hood, 2016; Zhao et al., 2021). Koo, Cho, and colleagues showed that treadmill running elevates p-AMPK, LC3-II, SIRT1, PGC-1α, and Parkin while reducing α-syn deposition, consistent with multivalent enhancement of mitophagy (Koo and Cho, 2017).

At downstream execution steps, exercise promotes TFEB activation to augment autophagy–lysosome degradation. In resting cells, TFEB is retained in the cytosol by mTORC1-dependent phosphorylation; exercise-induced AMPK and Ca^2+^ signals favor TFEB dephosphorylation and nuclear translocation, upregulating LAMP1/LAMP2/CTSD and facilitating autophagosome–lysosome fusion and cargo breakdown (Li et al., 2025; Mansueto et al., 2017). In α-syn transgenic mice, sustained physical activity increases PPARα and TFEB expression/nuclear localization and upregulates LAMP2 and CTSD, which—together with macroautophagy and chaperone-mediated autophagy (CMA)—promote α-syn clearance (Erlich et al., 2018). It is important to note that mitophagy primarily targets damaged mitochondria; improvements therein can indirectly suppress protein deposition by lowering ROS/inflammation, whereas direct removal of α-syn aggregates is mainly mediated by macroautophagy and CMA (Sala et al., 2016).

Summary. Exercise reinforces mitophagy at initiation (AMPK–ULK1), network maintenance (SIRT1–PGC-1α), and terminal degradation (TFEB), thereby facilitating clearance of α-syn–linked pathological substrates and supporting anti-aggregation effects in PD. Future work should dissect temporal dynamics and crosstalk among these pathways to optimize exercise-based strategies for α-syn removal and neuroprotection.

### 5.3 How exercise-activated mitophagy attenuates neuroinflammation in PD

Neuroinflammation is integral to PD progression (Zhu et al., 2025). Dysfunctional mitochondria chronically release mitochondria-derived damage-associated molecular patterns (mtDAMPs)—including ROS and mitochondrial DNA (mtDNA)—which activate the NLRP3 inflammasome and NF-κB/MAPK signaling, driving microglial hyperactivation and proinflammatory cytokine release (Deus et al., 2022; Mishra et al., 2025; Yan et al., 2020; Yu et al., 2025). By selectively removing damaged mitochondria, mitophagy curtails the supply of mtDAMPs and is closely associated with resolution of inflammation (Zhong and Lemasters, 2018).

In MPTP and 6-OHDA models, sustained treadmill running not only improves Δψm and lowers intracellular ROS but also produces favorable inflammatory endpoints: reduced CD11b/Iba1 (microglial activation markers) in the substantia nigra, decreased NLRP3–ASC–caspase-1 activity and IL-1β/IL-18 release, and downregulation of p-NF-κB and p-ERK/JNK/p38 (Gao Y. et al., 2025; Tung et al., 2024; Wang W. et al., 2021; Zhao et al., 2072). These shifts coincide with enhanced mitophagy flux—LC3-II/p62 changes indicative of increased throughput—and stronger PINK1/PRKN-mediated tagging/clearance (Chakraborty et al., 2018; Li and Chen, 2019; Wu et al., 2018).

Causality is supported by the observation that pharmacological autophagy inhibitors (3-MA, bafilomycin A1) or genetic suppression of PINK1/PRKN blunt exercise-induced reductions in IL-1β/TNF-α and microglial activation; conversely, TFEB activation partially mimics exercise’s anti-inflammatory effects, whereas TFEB inhibition attenuates them (Zhao et al., 2023).

Summary. By boosting clearance of damaged mitochondria and lysosomal degradative capacity, exercise reduces persistent release of ROS/mtDNA and dampens inflammasome activity, microglial activation, and proinflammatory cytokines in PD models (Koo and Cho, 2017; Ni et al., 2025; Sui et al., 2024). The evidence supports an “exercise → mitophagy → anti-inflammation” axis to guide prescriptions aimed at inflammatory control.

### 5.4 How exercise-activated mitophagy ameliorates calcium dyshomeostasis in PD

Calcium dysregulation underlies DA-neuron vulnerability in PD. Chronic high firing and rhythmic activity increase intracellular Ca^2+^ amplitude and burden; impaired mitochondrial buffering leads to matrix Ca^2+^ overload, Δψm collapse, mPTP opening, and intrinsic apoptosis (Matuz-Mares et al., 2022). Mitophagy selectively removes mitochondria with defective Ca^2+^ handling and depolarized membranes, thereby curtailing Ca^2+^ toxicity and helping restore homeostasis (Borbolis et al., 2025). Exercise-induced energy and Ca^2+^ signals act as upstream triggers that enhance mitophagy at multiple stages and are associated with improvements in PD-related Ca^2+^ abnormalities.

Exercise-related energy and Ca^2+^ signals facilitate initiation: increased AMP/ATP activates AMPK, which phosphorylates ULK1 at Ser317/Ser777 to assemble the ULK1–Atg13–FIP200 complex. Activity-evoked Ca^2+^ transients engage CaMKKβ and calcineurin to potentiate initiation and link to downstream TFEB dephosphorylation/nuclear translocation (Guan et al., 2024; Hatsuda et al., 2023; Laker et al., 2017), enabling early recognition and sequestration of compromised mitochondria. In animal models, treadmill/endurance training strengthens these initiation events and coincides with reduced indices of mitochondrial Ca^2+^ overload and lower mPTP propensity (Gill et al., 2019).

The PINK1/Parkin pathway then targets “Ca^2+^-overloaded, depolarized” mitochondria: upon Δψm loss, PINK1 accumulates on the OMM, recruits Parkin, ubiquitinates OMM substrates, and directs organelles to degradation. In MPTP/6-OHDA models, regular treadmill exercise enhances PINK1/Parkin enrichment on mitochondria and subsequent clearance efficiency, reflected by fewer Ca^2+^-overloaded mitochondria, restored membrane integrity/Ca^2+^ buffering, and favorable LC3-II/p62 flux signatures (Li et al., 2021). Finally, exercise-induced TFEB activation improves terminal degradation and reduces retention of “Ca^2+^-overloaded” mitochondria, with upregulation of LAMP1/LAMP2 and CTSD promoting fusion and breakdown (Triolo et al., 2022).

Summary. By reinforcing AMPK–ULK1 initiation, PINK1/Parkin-mediated selection, and TFEB-driven terminal degradation, exercise upgrades recognition, removal, and disposal of “Ca^2+^-dysregulated” mitochondria; these multi-level effects correlate with reduced mPTP opening and restoration of Δψm and Ca^2+^ buffering (Borbolis et al., 2025; Gill et al., 2019; Li et al., 2021; Matuz-Mares et al., 2022; Triolo et al., 2022).

### 5.5 How exercise-activated mitophagy counteracts ferroptosis in PD

Ferroptosis—an iron-dependent, lipid-peroxidation-driven form of regulated cell death—has been implicated in PD (Do Van et al., 2016). Hallmarks include increased mitochondrial membrane density, cristae loss, and depolarization, underscoring tight links to mitochondrial dysfunction. Damaged mitochondria are both a major ROS source and a nexus for iron dyshomeostasis; in the absence of effective clearance, they seed chain-propagating lipid peroxidation and amplify ferroptotic drive (Dong et al., 2023). Emerging evidence suggests that exercise mitigates neuronal ferroptosis by enhancing mitophagy and removing mitochondria with dysregulated iron handling.

First, by activating the AMPK–mTOR–ULK1 axis, exercise promotes the initiation and throughput of mitophagy, enabling timely removal of ROS-rich mitochondria and interruption of early lipid-peroxidation cascades (Lee et al., 2020). In animal studies, exercise elevates LC3-II, reduces p62 accumulation, downregulates ferroptosis markers (e.g., ACSL4, 4-HNE), and preserves glutathione peroxidase-4 (GPX4) activity—collectively indicating mitigation of lipid peroxidation via autophagy activation (Zhang et al., 2025).

Second, PINK1/Parkin-mediated mitophagy plays a central role in exercise’s anti-ferroptotic effect. Upon depolarization, PINK1 accumulation and Parkin recruitment target mitochondria—often enriched in Fe^2+^ and producing excessive ROS—for degradation, thereby disrupting Fenton-driven oxidative cascades (Xu J. et al., 2025). Exercise enhances PINK1 and Parkin expression and mitochondrial translocation, accelerating removal of iron-dysregulated mitochondria and lowering susceptibility to ferroptosis.

Third, the PGC-1α axis is crucial for mitochondrial iron metabolism and antioxidant defense. Beyond biogenesis, PGC-1α regulates mitochondrial iron-transport proteins (e.g., mitoferrin-1/2, ABCB8) to maintain iron homeostasis (Halling and Pilegaard, 2020) and supports expression of antioxidants such as GPX4. Together with mitophagic clearance, this forms an integrated barrier against ferroptotic stress. In PD models, exercise upregulates PGC-1α and downstream targets (NRF-1, TFAM), restores membrane integrity, and reduces labile iron and lipid-peroxidation markers (Gerecke et al., 2010).

Summary. Exercise reduces neuronal susceptibility to ferroptosis through (i) AMPK–ULK1-driven augmentation of mitophagy flux, (ii) PINK1/Parkin-dependent removal of iron-overloaded mitochondria, and (iii) PGC-1α-mediated stabilization of mitochondrial iron handling and antioxidant capacity—supporting mitophagy-targeted anti-ferroptotic strategies in PD.

## 6 Conclusion and outlook

In sum, mitophagy—a linchpin of mitochondrial quality control and cellular bioenergetics—plays a central role in PD pathophysiology. Dysfunction of mitophagy is tightly connected to oxidative stress, α-syn aggregation, neuroinflammation, calcium dyshomeostasis, and ferroptosis. A substantial body of evidence shows that exercise enhances mitophagy by engaging AMPK/ULK1, PINK1/Parkin, SIRT1/PGC-1α, and TFEB. Through coordinated regulation of recognition, sequestration, and degradation, exercise efficiently clears dysfunctional mitochondria, reduces ROS and pathological protein accumulation, alleviates cytotoxicity and inflammation, and improves multiple facets of PD pathology.

Different modalities (e.g., aerobic and resistance training) offer complementary advantages in maintaining mitochondrial homeostasis, and clinical studies further substantiate improvements in antioxidant defenses and functional outcomes in PD. Nevertheless, key knowledge gaps remain regarding the molecular underpinnings of exercise-induced mitophagy—crosstalk between AMPK and PINK1/Parkin, the precise roles of SIRT3, DJ-1, irisin, and other modulators, and the temporal/ “dose–response” characteristics of autophagy activation.

Future work should leverage multi-omics and gene-editing tools in large, multi-center clinical studies to systematically evaluate the impacts of diverse exercise paradigms on mitophagy and PD phenotypes, and to explore synergies with nutritional and pharmacological interventions. Looking ahead, individualized, mitophagy-targeted exercise prescriptions hold promise as a non-pharmacological avenue for PD management and may provide a foundation for disease-modifying therapy—offering a practical framework for precision medicine in neurodegenerative disorders.
